# Assessing treatment outcomes among peer educators living with HIV in Kenya

**DOI:** 10.1371/journal.pone.0218774

**Published:** 2019-06-27

**Authors:** Joram Luke Sunguti, Appolinaire Tiam, Rose Masaba, Michael Waweru, Judith Kose, Justine Odionyi, Lucy Matu, Eliud Mwangi

**Affiliations:** 1 Elizabeth Glaser Pediatric AIDS Foundation, Nairobi, Kenya; 2 Elizabeth Glaser Pediatric AIDS Foundation, Washington DC, United States of America; The Ohio State University, UNITED STATES

## Abstract

**Background:**

People living with HIV (PLHIV) often face barriers in accessing quality and comprehensive HIV care, including stigma and discrimination, which results in poor retention and viral non-suppression. Peer-led interventions can help address these barriers. In Kenya, peer educators (PEs) are PLHIV who support other PLHIV to adhere to clinic schedules and antiretroviral medication uptake. In spite of their status as role models and their key role in supporting clients receiving HIV care and treatment, little is known about the characteristics and treatment outcomes of PEs themselves, specifically viral suppression.

**Methods:**

This is a retrospective descriptive analysis of program data on treatment outcomes of PEs engaged in active patient support activities between October 2010 and January 2017. All eligible PEs from 140 health facilities located in 23 counties of Kenya were included in the study. Data from 230 PEs were abstracted from the electronic medical records, patient files, and registers between June and August 2017. Study variables included key sociodemographic characteristics (sex, marital status, and age), duration on antiretroviral therapy (ART), WHO clinical staging, baseline CD4 count, current antiretroviral regimen and uptake of isoniazid preventive therapy (IPT). The outcome variable was viral suppression, defined as a viral load <1000 copies/ml.

**Results:**

Overall, 173/230 (75%) of the PEs were female, 144/230 (63%) were married, and median age (LQ, UQ) was 38.5 (33.0, 42.0) years. The PEs had been on ART for a median (LQ, UQ) duration of 76.0 (37.0, 105.0) months. Six months IPT completion was high at 97%. Of the 222 (97%) PEs with an up-to-date viral load taken within the last one year, 211 (95%) were virally suppressed.

**Conclusion:**

Our study showed that peer educators actively engaged in patient support activities have achieved high viral suppression rates.

## Introduction

In July 2016, the Kenyan Ministry of Health (MOH) rolled out the HIV Test and Treat guidelines, in line with the WHO recommendations [1]. Some notable advantages of antiretroviral therapy (ART) for people living with HIV (PLHIV) include improved quality of life, reduced HIV transmission, decreased morbidity and mortality [2–3]. In Kenya, there are 1.5 million people living with HIV, one million of whom are on ART with a 12-month retention rate of 81% and a viral suppression of 75% [4]. The MOH aims to achieve greater than 90% ART coverage in line with the UNAIDS 90-90-90 strategy. With the implementation of Test and Treat, the number of PLHIV actively taking lifelong ART is expected to increase. Optimization of adherence and retention within this population is important, as studies have shown that poor adherence is associated with virological failure [5–6].

PLHIV often face barriers in accessing quality and comprehensive HIV care, including stigma and discrimination, which results in poor retention [7]. Peer-led support can help address these barriers, and has been used in management of chronic diseases such as diabetes [8], smoking [9] and cancer [10]. Some notable benefits of peer-led initiatives include improved patient outcomes such as glycemic control, increased HIV testing and retention in care [11–14].

Active engagement of PLHIV as peer educators (PEs) responds to the need for a multidisciplinary approach. PEs utilize their life experiences to support prevention, adherence and retention among other PLHIV. In HIV programming, PEs are PLHIV who support other HIV-positive patients to adhere to medication and clinic schedules. Requirements of a PE in Kenya include: self-disclosure, ability to read and write, ART enrollment and participation in a psychosocial support group at one facility, demonstration of good adherence to ART, and acting as a good example for other PLHIV. Table 1 highlights the roles and responsibilities of PEs.

**Table 1:** Roles and responsibilities of PEs (adapted from the Kenya task shifting guidelines).

Management of patient appointment
• Updating of the appointment card and diary • Sending SMS reminders
Defaulter tracing and management
• Compilation of daily missed appointments • Updating of the defaulter tracing register • Defaulter tracing by use of phone and/or home visits
Adherence counselling
• Conducting psychosocial assessment • Conducting health education and patient literacy sessions • Conducting routine and booster adherence counselling
Psychosocial support
• Establishment of psychosocial support groups (PSSG) and ensuring that they are functional • Maintaining and updating of PSSG records • Conducting Positive Health Dignity and Prevention (PHDP) sessions
Referral and linkages
• Referral of clients to service points within the facility • Maintenance of the community-facility referral directory • Documentation and tracking of facility-facility/community referrals
Record keeping
• Filing of patient records • Preparation of timely monthly reports on patient appointment, PHDP and PSSG

Several studies in Kenya have evaluated the role of PEs in HIV service delivery. In one study, PEs were recruited to help improve HIV diagnosis, linkage to ART, and adherence and retention. In the first year, over 90% of eligible, in-patient clients were provided with pre-test counseling and referred for testing [15]. In Homa Bay County in Western Kenya, involvement of adolescent PEs led to more than 97% linkage and more than 90% early retention among adolescents on ART [16]. PEs have also been shown to improve adherence and retention in men’s adherence clubs [17,18]. While PEs form an essential component of psychosocial support within the health system, their own health is as important as the health of those they support. PEs are an important piece of the puzzle in improving HIV treatment outcomes among PLHIV and so it is important to ensure they have good health. Despite existing literature highlighting the importance of PEs, studies have not focused on their well-being as they continue to be actively involved in patient. To date, studies have not explored viral suppression among PEs supporting PLHIV in Kenya. Due to the limited availability of data in this area, we aimed to determine treatment outcomes among PEs, specifically viral suppression.

## Materials and methods

### Study design

We conducted a retrospective descriptive analysis of program data on treatment outcomes of PLHIV who are engaged in active patient support activities. The PEs were drawn from 140 health facilities located in 23 counties where Elizabeth Glaser Pediatric AIDS Foundation (EGPAF) supports HIV service delivery. Data were collected on all PEs in the 140 health facilities.

### Participants

For this study, we abstracted data on HIV-positive PEs who had been selected and engaged as PEs between October 2010 and January 2017. Data from 230 PEs were abstracted from the electronic medical records, patient files, and registers between June and August 2017. To be included in the study, the PEs had to be HIV-positive, enrolled on ART for more than six months, and providing psychosocial support to other PLHIV. In this study, we included all PEs who met the eligibility criteria and whose data were available.

### Measures

Patient level data were abstracted from the electronic medical records, patients’ files and registers using a standardized data abstraction tool. Predictor variables included key sociodemographic characteristics (sex, marital status, and age), duration on ART, WHO clinical staging, baseline CD4 count, current ART regimen and uptake of isoniazid preventive therapy (IPT). Advanced HIV disease was defined by CD4 count less than 200 cells/ml. One was considered to have received IPT if they completed 6 moths of IPT as recommended in the national guidelines. The primary outcome variable was viral suppression. We collected viral load results that were up-to-date at the time of data collection. Up-to-date viral load was defined as a viral load taken within the last twelve months with available results. We defined HIV viral suppression as the latest HIV RNA viral load value of less than 1,000 copies/ml as defined in the 2016 Kenyan MOH guidelines [1].

### Laboratory methods

As part of routine program, viral load samples were collected as whole blood, dried blood spots or plasma and submitted to the processing laboratories which analyzed the viral load using either *Abbott* RT-PCR (Real-time polymerase chain reaction) (Rungis, France) for HIV-RNA or Roche *Amplicor* RT-PCR (Maylan, France). These platforms receive and process samples from the 140 health facilities included in the study. According to Kenyan ministry of health national guidelines, the first viral load is usually done after 6 months on ART followed by an annual viral load from the time of ART initiation. Viral suppression is defined as a viral load less than 1000 copies/ml.

### Analysis

Continuous data were described through median and percentiles. Categorical data were described-through use of proportions. To assess differences in clinical and sociodemographic characteristics between men and women, a two-sample Z-test was used to estimate differences in sample proportions while the Mann-Whitney U test was used for continuous variables. All reported p-values were two-sided and a p-value <0.05 was considered statistically significant. Statistical analysis was conducted using the IBM SPSS software version 20.0 for Windows (IBM, Chicago, IL, USA).

### Ethical considerations

The study protocol was reviewed and approved by the Kenyatta National Hospital Ethics Review committee in Kenya and the Chesapeake (Currently Advarra) IRB in the USA. A waiver of informed consent was received for this analysis.

## Results

**Fig 1:**
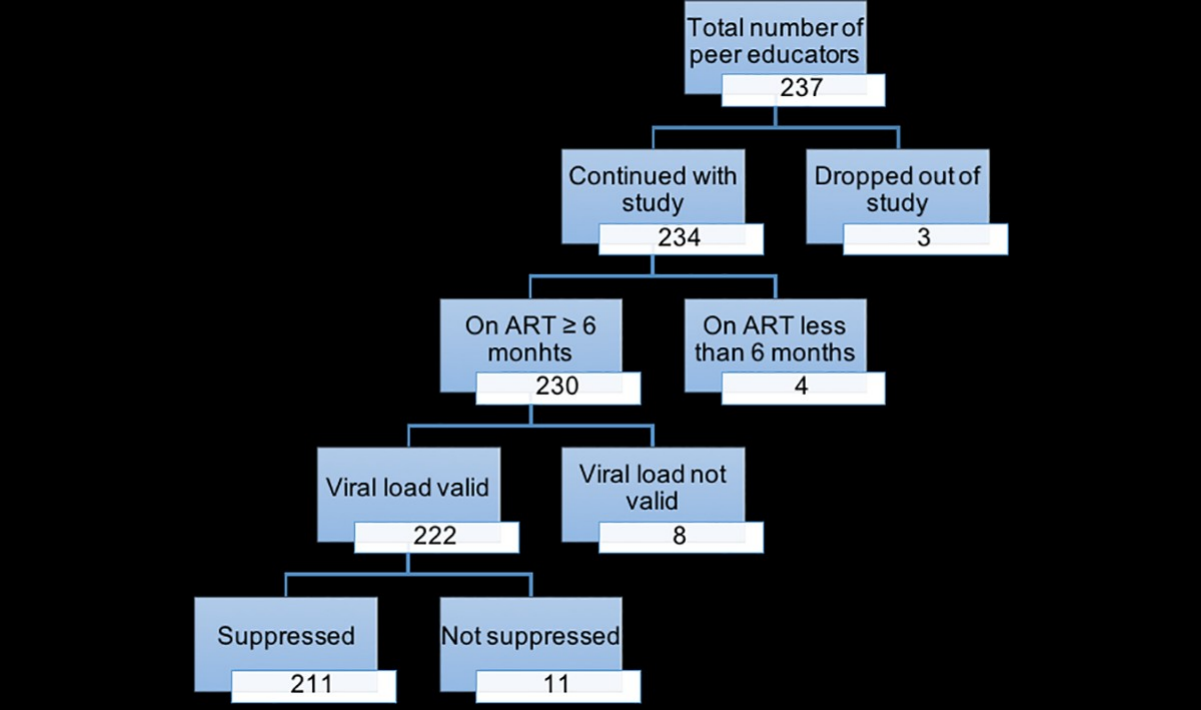
Participant enrollment into the study.

Of the 237 peer educators initially engaged in patient support in the 140 health facilities, three had dropped out of their job as peer educators and were not active in care in the three facilities where they had initially enrolled. Due to unavailability of their recent data within patient records, we excluded them from the study. At the time of study enrollment, four of the 234 PEs had been on ART for less than six months and were excluded from the study, as they were not yet eligible for the viral load test. The number of PEs included in the final analysis was 230 (Fig 1).

Overall, 173/230 (75%) of the PEs were female and 144/230 (63%) were married (Table 2). The overall median age (LQ, UQ) was 38.5 (33.0, 42.0) years. Male PEs were marginally older with a median age (LQ, UQ) of 42.0 (36.0, 48.0) years compared to female PEs, whose median age (LQ, UQ) was 37.0 (32.0, 42.0) years. The PEs had been on ART for a median (LQ, UQ) duration of 76.0 (37.0, 105.0) months. At the time of enrollment into HIV care, 61/230 (27%) PEs had advanced HIV disease. The number of PEs on the various ART regimens were as follows: TDF/3TC/EFV 102 (44%), AZT/3TC/NVP 75 (32%), TDF/3TC/NVP 30 (13%), AZT/3TC/EFV 12 (5%) and other 11 (5%). The majority of the PEs (97%) had completed six months of IPT. Of the 222 (97%) PEs with an up-to date valid viral load, 211 (95%) were virally suppressed. Of note, male PEs were more likely to be married, older, and with more advanced disease than females.

**Table 2:** Sex-specific characteristics of 230 HIV-positive PEs in 140 sites in Kenya.

Characteristics	N (Column %) or Median (LQ, UQ))			P value*
	Total	Male	Female	
Marital status				
Married	144 (63%)	53 (93%)	91 (52%)	**<0.001**
Divorced	8 (3%)	1 (2%)	7 (4%)	0.27
Single	9 (4%)	3 (5%)	6 (4%)	0.75
Widow/Widower	69 (30%)	0 (0%)	69 (40%)	**<0.001**
Median age in years (LQ, UQ)	38.5 (33.0, 42.0))	42.0 (36.0, 48.0)	37.0 (32.0, 42.0))	**0.002**
Median duration on ART in months (LQ, UQ)	76.0 (37.0, 105.0)	76.0 (49.5, 104.0))	76.0 (35.5, 105.0)	0.38
WHO staging at enrollment				
Stage 1	71 (31%)	15 (26%)	56 (32%)	0.39
Stage 2	73 (32%)	14 (25%)	59 (34%)	0.21
Stage 3	78 (34%)	26 (46%)	52 (30%)	**0.03**
Stage 4	8 (3%)	2 (3%)	6 (4%)	0.18
Mean CD4 in cells/mm3 (SD)	350 (260)	321 (254)	359 (4)	0.33
CD4 category				
<200	61 (27%)	19 (33%)	42 (24%)	0.18
200+	169 (73%)	38 (67%)	131 (76%)	0.18
Current ART regimen				
TDF/3TC/EFV	102 (44%)	23 (40%)	79 (46%)	0.43
AZT/3TC/NVP	75 (33%)	20 (35%)	55 (32%)	0.68
TDF/3TC/NVP	30 (13%)	9 (16%)	21 (12%)	0.43
AZT/3TC/EFV	12 (5%)	4 (7%)	8 (5%)	0.57
Other	11 (5%)	1 (2%)	10 (5%)	0.33
Completed 6 months of IPT				
Yes	219 (95%)	55 (96%)	164 (95%)	0.76
No	11 (5%)	2 (4%)	9 (5%)	0.76
Viral load taken within the last 12 months				
Yes	222 (97%)	54 (95%)	168 (97%)	0.48
No	8 (3%)	3 (5%)	5 (3%)	0.48
Virally suppressed (VL < 1000 copies/ml)				
Yes	211 (95%)	48 (89%)	163 (97)	**0.02**
No	11 (5%)	6 (11%)	5 (3%)	0.4

11% of the men were unsuppressed compared to 3% of the women (Table 2). We further evaluated univariate correlates of viral suppression, as shown in Table 3 below.

**Table 3:** Univariate correlates of viral suppression among 222 HIV-positive PEs in 140 sites in Kenya.

Characteristic	Viral suppression: N (Row %)		
	Suppressed	Unsuppressed	RR for unsuppression (95% CI)
Sex			
Male (Ref)	48 (89%)	6 (11%)	-
Female	163 (97%)	5 (3%)	0.9 (0.8–1.0)
Marital status			
Married (Ref)	128 (93%)	9 (7%)	-
Not married*	83 (98%)	2 (2%)	0.9 (0.9–1.0)
Age (years)			
<30	23 (82%)	5 (18%)	-
30–39	92 (98%)	2 (2%)	0.8 (0.7–1.0)
40–49	79 (98%)	2 (2%)	0.8 (0.7–1.0)
50+	17 (89%)	2 (11%)	0.9 (0.7–1.2)
Duration on ART (months)			
<36	44 (96%)	2 (4%)	-
36–71	55 (96%)	2 (4%)	1.0 (0.9–1.1)
72+	112 (94%)	7 (6%)	1.0 (0.9–1.1)
WHO staging at enrollment			
Non-advanced disease (WHO stage 1 and 2) (Ref)	132 (96%)	6 (4%)	-
Advanced disease (WHO stage 3 and 4)	79 (94%)	5 (6%)	1.0 (0.9–1.1)
Baseline CD4 (cells/mm3)			
<200	53 (90%)	6 (10%)	-
200+	158 (97%)	5 (3%)	0.9 (0.8–1.0)
Current ART regimen (Backbone)			
TDF based (Ref)	122 (95%)	6 (5%)	-
AZT based	82 (96%)	3 (4%)	0.9 (0.9–1.0)
Current ART regimen (Tail)			
EFV-based (Ref)	105 (96%)	4 (4%)	-
NVP-based	99 (95%)	5 (5%)	1.0 (0.9–1.1)
Received IPT			
Yes	202 (95%)	11 (5%)	-
No	9 (100%)	0 (0%)	-

*Includes single, divorced and widow/widower

## Discussion

In this study, we found that PEs had high rates of viral suppression. Of note is that this unique population has achieved the UNAIDS/WHO target of the third 90. To the best of our knowledge, limited studies have specifically evaluated viral suppression among PEs in Kenya and elsewhere as a specific patient population. We found the proportion of viral suppression reported within PEs to be higher than the overall viral suppression among adults living with HIV. Several studies in Kenya and our region report a viral suppression among general adult patient population ranging from 37% to 93% [4, 19–21].

The role of peer-led interventions in health care is gaining more interest. In one study, there was improved ART adherence among HIV positive clients randomized to receive peer-led adherence intervention [14]. Other documented benefits of peer-led interventions include increased HIV testing among people who inject drugs [12] and reduced plasma fasting glucose among people living with diabetes [11]. Peers share social networks and life experiences and this can increase acceptability of peer-led interventions. In two systematic reviews evaluating the efficacy of peer-led interventions, there was demonstrated benefits in some self-reported attributes such as HIV knowledge, attitudes and cognitions, sexual risk behavior, substance use and processes of care but none in biological and clinical outcomes [22,23]. Despite documented benefits of peer-led interventions within various healthcare settings, studies have mostly focused on the beneficiaries rather than their peer providers. This study specifically focuses on the outcomes of peer educators to determine their outcomes as they support other PLHIV. This in turn will inform provision of quality HIV care and treatment to this unique subset.

In our study, more women were PEs compared to men. This is reflective of the proportions of patients accessing HIV care in our clinics where about 67% of these are women. Men are possibly still under-represented. Most of the patients in the study were still on first-line ART and yet were virally suppressed, despite being a mature cohort that had been on ART for a prolonged period of time. With good adherence within this unique patient group, viral suppression can be achieved even with prolonged duration on first-line ART.

Similar to other studies in Kenya and elsewhere, female PEs were found to have higher suppression rates than males [24,25]. This finding can be attributed to women’s health seeking behavior. Female patients tend to follow medical recommendations better than male patients. A study in India showed that due to social norms, women prefer long-term treatment approaches and engagement with health providers thereby making them resilient [26]. In a study in the United Kingdom, it was found that women consulted and adhered better to clinical recommendation because they had more contact to the health system while growing due to the obstetric needs [27]. It must however be noted that overall, viral suppression among men in our study population was higher than in any previous reported data for men within sub-Saharan Africa, where men had a less than 89% viral suppression [28–30]. The relatively high suppression among this group of men is likely part of the overall improved suppression that was seen with all the peer educators.

While it is possible that involving PLHIV as peer educators may lead to worse outcomes among them due to the stress associated with work in addition to managing their own care, this was not noted in this study. Interesting, majority of the PEs had achieved viral suppression despite the fact that more than a quarter of them were enrolled on ART with advanced disease (CD4 < 200 or WHO stage 3 and 4). Several factors may have contributed to high viral suppression among PEs. PEs have the benefit of accessing patient literacy materials and working in an environment where adherence messages are being reinforced. They are also unlikely to miss appointments since they actually work in the facilities where they access their care and since they remind other patients of their appointment keeping, this may influence their own appointment keeping as role models. They are more likely to receive closer follow-up from the clinical team, and are more likely to have disclosed their status, which has likely led to better psychosocial support. Thus, when PLHIV are actively involved in their own care and that of other patients, this strengthens their adherence on ART and retention in care. Consequently, they will achieve viral suppression despite sociodemographic and clinical characteristics that generally predispose PLHIV to non-suppression.

However, it is important to note that, there may be inherent factors (character, experiences, family support, etc.) that make PEs good patients in the first place. It is possible that they were selected to be PEs by virtue of the fact that they are already good patients. Better viral suppression among PEs could therefore be explained by the fact that PEs may be a selected group (either self-selected or selected by the treatment programs) that are predisposed to have high adherence. Also, the act of being a PE may result in higher adherence. This study was not able to study this directly, but recognizes that these factors may have contributed to the findings. Qualitative studies would help researchers explore perspectives that make PEs more successful at achieving viral suppression.

A limitation of this study is that study findings are not generalizable to other PLHIV who are not PEs. Generally, PEs have better psychosocial support, have less self-stigma, and were selected to be PEs based on existing desirable outcomes. Therefore, it is difficult to conclusively delineate whether their successful viral suppression reported in this study is correlated to their work as PEs specifically or not. Another limitation is that, since most participants were virally suppressed, the sample size became too small to analyze all the possible factors associated with viral suppression, given the small number of those not suppressed.

Our study showed that PEs have high viral suppression rates. Longitudinal studies would help researchers and program implementers to understand further the reasons for better outcomes among PEs.

## Supporting information

S1 Peer Educator Dataset (CSV)
